# A clinical evaluation and acceptability study of the innovative SurePulse VS wireless heart rate monitor across the neonatal journey

**DOI:** 10.3389/fped.2024.1355777

**Published:** 2024-05-17

**Authors:** Jennifer Peterson, Clare Jennings, Ajit Mahaveer

**Affiliations:** ^1^ Faculty of Biology, Medicine and Health Sciences, The University of Manchester, Manchester, United Kingdom; ^2^ Neonatal Intensive Care Unit, St Mary’s Maternity Hospital, Manchester University NHS Foundation Trust, Manchester, United Kingdom

**Keywords:** neonates, resuscitation, wireless monitoring, family integrated care, monitor

## Abstract

**Background:**

The SurePulse vital signs (VS) device is an innovative wireless heart rate monitor designed for neonatal patients. This study evaluates the application of SurePulse VS technology in clinical practice.

**Methods:**

Data were collected about the quantitative metrics of the device itself when deployed on real infants and qualitative feedback from perinatal professionals and parents regarding their experiences using this novel technology.

**Results:**

This study recruited 101 infants and achieved target completion rates of 101 healthcare professional (HCP) and 51 parent questionnaires over the seven-month study period. The SurePulse device was deployed across a range of gestational ages (34–39 weeks) and birth weights (1.8–3.5 kg). Device deployment was performed across a range of clinical environments, with 51% of deployments at delivery and 47% within the neonatal unit. The data show clinically acceptable timings from device deployment to heart rate signal acquisition [median 20 s (IQR 15–76 s)]. HCP feedback rated SurePulse monitoring as “Always” or “Mostly” reliable in 80% of cases. Parental feedback reported that having the SurePulse device was reassuring, convenient and beneficial to them. These positive comments were reflected across device deployment in the delivery room and within the neonatal unit.

**Conclusions:**

The study findings show that the SurePulse device has potential to be a significant advancement in the way neonatal patients are monitored in a variety of post-delivery circumstances. This study has demonstrated that the SurePulse device has utility throughout the neonatal journey, enabling accurate heart rate monitoring in a manner that promotes parent-infant contact and bonding.

## Introduction

**Figure 1:**
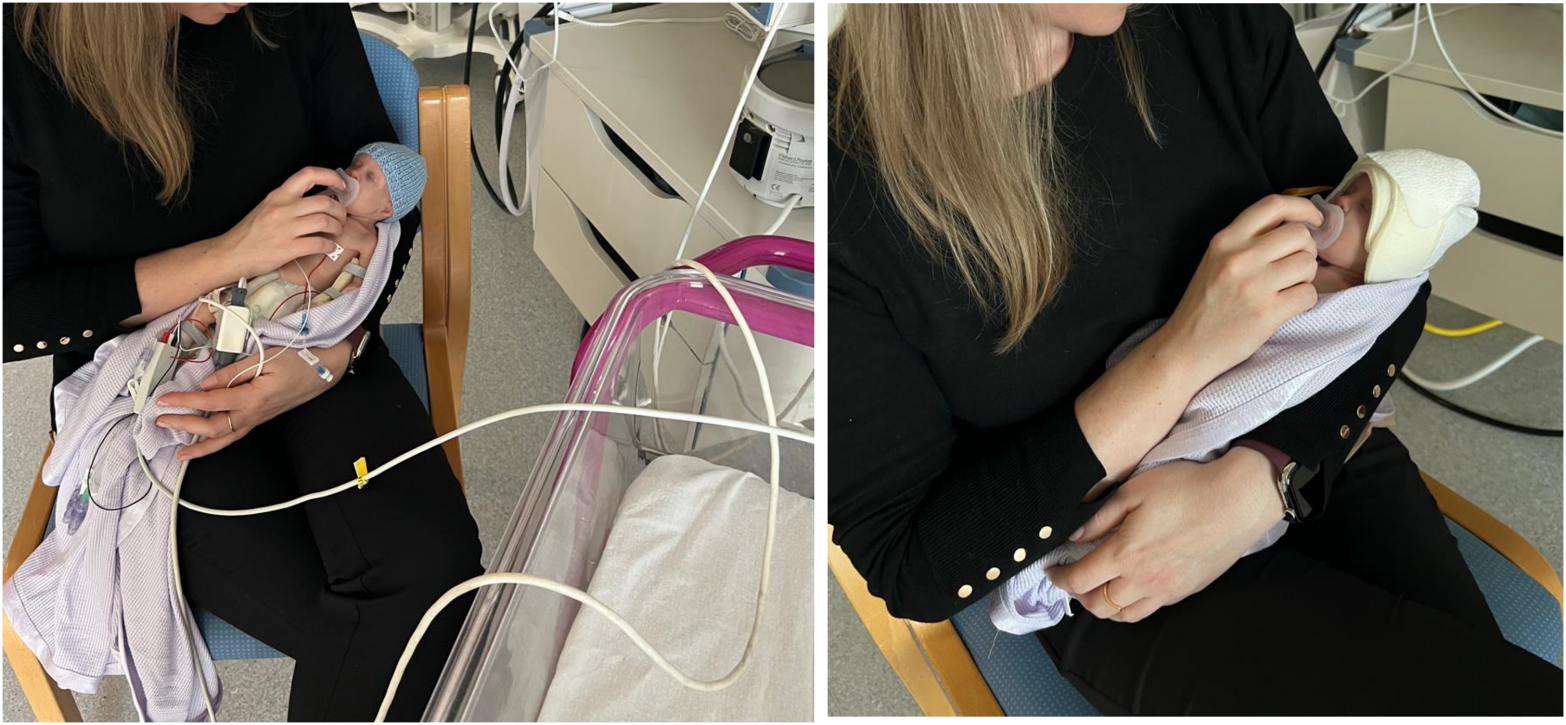
Wired vs. Wireless Monitoring. **Standard Wired Monitoring (left):** Infant attached to numerous wires. Tethered to cot-space. Parent having to manage wires alongside focusing on baby. **SurePulse Wireless Monitoring (right):** No wires. Parent can hold baby in the most comfortable position and can be repositioned easily as needed. Parent focus can be entirely on their baby.

Wired monitoring systems can cause physical and psychological barriers to the implementation of effective family-integrated care and parent-infant bonding (1). Family-integrated care (FIC) research demonstrates increasing evidence that integration of parents into their infant's care leads to improved outcomes for both infant and parent. Studies evaluating the impact of FIC show better outcomes across a range of domains, such as improved infant nutrition with increased weight gain, exclusive breastfeeding rates and decreased parental stress and anxiety in the FIC group (2). Parents should be enabled to feel comfortable and confident when handling their baby and/or providing cares. At present, most devices for infant monitoring involve wires between the sensor attached to the baby and the monitor screen. The presence of multiple wires attached to the baby can negatively impact the ability and confidence of parents to touch and hold their infant (2). There can be a perceived association between the presence of medical device wires and the severity of the infant's condition which may compound parental stress. The presence of monitoring wires may also increase concerns about the feasibility of performing simple tasks, such as getting baby out for a cuddle (Figure 1), and this can erode parent confidence (3). Parents may be deterred from wanting to participate in cares and cuddles due to the potential for unintended impacts on clinical care through monitoring wires becoming tangled; for example, tangled wires causing respiratory support to be compromised or an intravenous line to be dislodged. Additionally, in the preterm population, multiple sensors and device wires can cause skin irritation and breakdown, which is both painful and a risk for infection (4, 5).

Despite these issues monitoring is vital to ensure the safety of infants at birth and in cases where ongoing medical intervention on the neonatal unit is required. At delivery, rapid and accurate heart rate monitoring is crucial for guiding resuscitation interventions for compromised neonates (6). Current wired monitoring methods, such as electrocardiogram and pulse oximetry, can have delays in signal acquisition which can prolong assessment of heart rate (7). If admitted to the neonatal unit, infants born prematurely and/or with medical or surgical conditions require mandatory monitoring to ensure their safety and guide their treatment. Additionally, minimally invasive monitoring during post-delivery skin-to-skin and breastfeeding has the potential to improve safety for infants on the postnatal wards (8). Whilst unexpected postnatal collapse is a rare event, it can have devastating consequences due to the high risk of mortality, and lifelong morbidity for survivors (9, 10). Wireless monitoring in this context would allow signs of compromise, such as a sustained decrease in heart rate, to be detected and acted upon, without the visual and physical restrictions of wires impacting bonding between parent and baby.

### The role of wireless monitoring

Devices used to monitor infants must be safe and effective. Given advancing technologies, there is increasing interest in developing monitoring interfaces which are more infant, parent and professional friendly. This is reflected in an article by Batey, et al. (2022) which reports that development of wireless vital sign monitoring was ranked by perinatal professionals as a top three priority goal for the future of neonatal technologies (11).

The SurePulse vital signs (VS) wireless neonatal monitoring device (Nottingham, UK) is an innovative new technology which provides accurate and reliable monitoring with the additional advantage of a wireless interface (12). The SurePulse device utilises a forehead reflectance photoplethysmography sensor built into the brow band of a soft, adjustable cap. Application of a cap in the delivery room is standard practice to facilitate normothermia in the post-delivery period. Therefore, this device is easily integrated into normal practice and aligns well with the Newborn Life Support algorithm (6). The cap comes in a variety of sizes (extra small, small, medium, large and extra large) to fit a range of gestational ages from 23-weeks gestation to post-term infants. Each size of cap can be further adjusted to ensure a snug but comfortable fit for the individual infant's head shape. The sensor in the browband should be aligned over the infant's right eyebrow to optimise monitoring success (Supplementary Figure S1). The current iteration of the SurePulse device has been designed for heart rate monitoring only. The device does not currently provide oxygen saturation (SpO2) or temperature detection. It works by inserting a wireless module into the cap cradle which connects wirelessly (via Bluetooth) to the heart rate monitoring display screen. This can be placed anywhere in the room to suit the HCP. However, for optimal readings the monitoring screen should ideally be placed in a direct line of sight to the infant. For example, in the context of a compromised infant at birth, the monitor can be placed at the end of the resuscitaire (near the foot end of the infant) where the communication path between the SurePulse VS module and the monitoring screen are in a direct line and where the clinician has a clear view of the detected heart rate on the monitor screen. Bluetooth disconnection between the module and display screen was not an issue while conducting the study. However, if this were to occur, the healthcare professional would simply place the module back into the display screen dock, allow the connection to reestablish and then detach and reinsert the module back into the hat.

## Methods

This study primarily aimed to evaluate the application of SurePulse technology in clinical practice, collecting data on quantitative metrics of the device itself when deployed on real infants and qualitative feedback from perinatal professionals regarding their experiences using this novel technology. The secondary aim was to gather parental feedback about their perception of the SurePulse device functionality and comfort in relation to their baby.

The study was conducted in a large tertiary maternity unit which delivers 13,000 infants per year (13), following ethical approval (London—Brighton & Sussex Research Ethics Committee 21/PR/0293). The study was conducted between August 2021—February 2022 inclusive and recruited 101 infants. Data were gathered through device downloads, via USB, from each individual infant device deployment and completion of 100 healthcare professional (HCP) questionnaires. Parent feedback was gathered through written questionnaires administered at the end of the device deployment period. The aim was to gather 20–40 parent responses.

Where parents had provided consent prior to the delivery, the infant had the SurePulse cap applied at birth which allowed heart rate recording during the immediate post-birth transition of the infant from fetal to neonatal circulation. This occurred variably on the resuscitaire or during skin-to-skin with the mother depending on the condition of the infant at birth. Infants were also recruited post-birth within the neonatal unit from the high dependency and special care areas. These infants were clinically stable and had the SurePulse cap applied at a convenient time for the infant and their parent. Neonatal unit recruits often had the SurePulse device deployed around periods of care and/or cuddles with parents. In all device deployments the infants continued to receive standard monitoring alongside the SurePulse monitoring.

### Training

As this was a new medical device staff training was paramount to ensure patient safety and study success. Prior to the study launch the SurePulse development team delivered formal one-hour training sessions for perinatal staff covering use of the study device at deliveries, both in delivery rooms and in theatre. The neonatal research team received additional training to allow them to be SurePulse trainers able to deliver individual same-day training sessions and refreshers to perinatal clinical staff.

## Results

The study recruited 101 infants and achieved completion of 101 healthcare professional questionnaires and 51 parent completed questionnaires over the seven-month study period. A range of infants were recruited with a median gestational age of 37 + 3 weeks (IQR 34 + 1–39 + 4 weeks) with a median birth weight of 2.5 kg (IQR 1.8–3.5 kg). Within our study cohort, 51% of device deployments were at deliveries (obstetric theatres (31%) and delivery suite or midwifery-led unit (20%)). In 47% of cases the device was utilised for infants being cared for on the neonatal unit. The remaining two infants did not have the location of device deployment recorded.

Overall feedback from HCP's and parents showed significant support for the device indicating that wireless monitoring would be beneficial in a variety of clinical circumstances (Figures 3, 4).

i. Recruitment

In order to achieve recruitment of 101 infants, our team approached 183 parents of which 82 declined to participate. Given that this study was non-invasive and required only a short participation period from the family, this was a higher decline rate than anticipated. Whilst reasons for declining were not formally sought, informal feedback freely stated by parents was that having to read and consider participation in the study whilst they were in labour or awaiting the birth of their child was excessively burdensome. This provides some explanation for the higher rates of device deployment for infants already on the neonatal unit and for births via elective caesarian section compared to delivery suite births.

ii. Healthcare Professional Feedback

**Figure 2:**
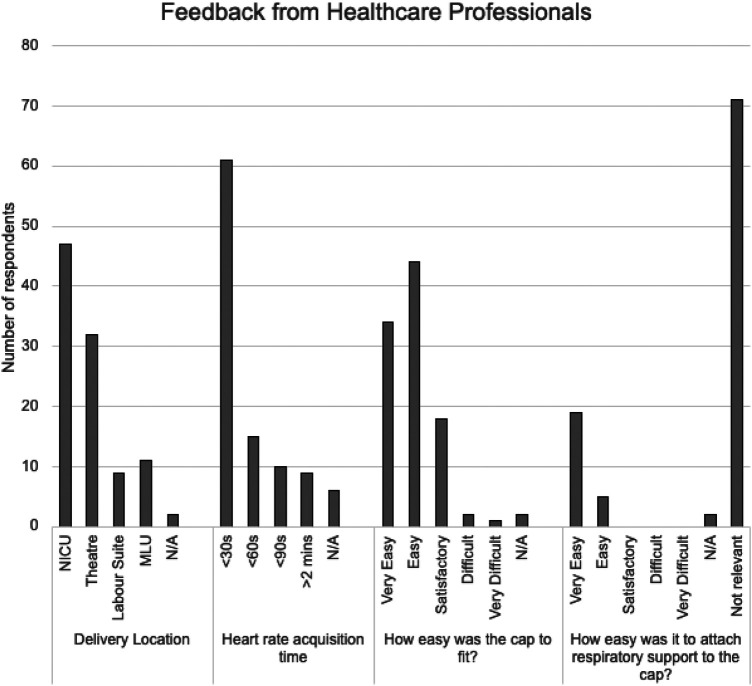
Summary of Feedback from Healthcare Professionals. (NICU = neonatal intensive care unit; MLU = Midwifery Led Unit; N/A = Not applicable).

Data from the healthcare professional questionnaires show that the device was deemed useful and easy to use in the majority of cases (Figure 2). A key element of ensuring accurate SurePulse monitoring is correct selection and application of the cap. This is necessary to optimise sensor placement, which should be over the right eyebrow and comfortably but firmly held in place via the band of the cap. HCP's rated ease of fitting the SurePulse cap as either “Easy” or “Very Easy” in 77% of cases. Eighteen percent of professionals rated the fitting process as “Satisfactory”. Only two HCP's reported this was “Difficult” and one additional user reported the fitting as “Very Difficult”. Where there were issues with fitting the cap, the main concern from HCP's was the shape of the cap and this not aligning well with the head shape of the infant. The cap material is similar to the crepe texture of a bandage. The current design relies on the HCP securing each section of the cap together around the infant's head to try and give a snug fit (Supplementary Figure S1). However, due to the design and material the cap is problematic to fit around infants with an elongated head shape at birth or infants with a pronounced occiput. Qualitative feedback showed multiple HCP's (10 written comments) requesting for a change in the design of the cap to better address these issues. An additional concern from HCP's was regarding the design of the module attachment. In order to ensure the cap was comfortable and to minimise risk of injury to the infant, the plastic wireless module is inserted into a module cradle connected to, but not forming part of the cap (Supplementary Figure S1). This means that the module then dangles by the side of the baby's head. This is cumbersome during skin-to-skin, cuddles or moving the baby. Professionals reported they would have preferred a simple mechanism to secure the module to the cap. Many staff utilised the additional piece of fabric which came with the cap—originally intended to attach respiratory support if required—and used this to attach the module to the cap during cuddles (Supplementary Figure S1). A more definitive method should be included in future iterations of the cap design.

Professionals rated the study device monitor as “Always” or “Mostly” reliable in 80% of cases (44 and 37 HCP's respectively). In five cases HCP's rated the device as “Not Reliable”. These five cases were split between the delivery room and NICU. HCP's in these cases reported issues with fitting the cap, particularly whilst delivering airway support to the infant. Written comments highlighted that the cap could be improved by using an elasticated design in future.

Of the 101 infants recruited, 26 required respiratory support whilst having SurePulse monitoring. In 19 of these cases professionals reported that it was “Very Easy” to attach respiratory support to the cap and an additional 5 rated this as “Easy”. Healthcare professional reported that they would prefer the study device to be able to record oxygen saturations and temperature alongside heart rate monitoring. This was an important factor to HCP's as without these additional parameters the infant would continue to require a wired saturations monitor and therefore, would not benefit from the advantages of wireless monitoring.

Training was rated as satisfactory by 96% of HCP's. The remaining 4% did not comment on the quality of their training session.

iii. Parent Feedback

**Figure 3:**
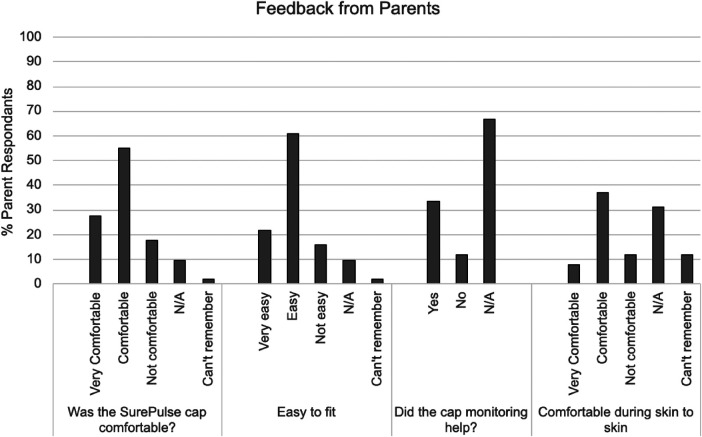
Summary of Feedback from Parents. (N/A = Not applicable).

The study achieved successful completion of 51 parent questionnaires, exceeding the original study goal. The majority of parents reported that the SurePulse caps were comfortable and easy to fit. There were 31 parents who had the study device deployed whilst having skin-to-skin and 74% of this cohort report the device was comfortable (Figure 3).

The majority of infants recruited to this study were physiologically stable. This may explain the reason that the majority of parents (67%) did not rate whether or not the study device was useful. For parents who did rate the usefulness of having this wireless monitoring, the majority felt that it was useful (74%).

Parents commented that the current cap design could be optimised by having a different material that had more stretch and was better able to fit different head shapes. Comments from parents highlight that there is an issue with the current cap slipping when the baby is being moved and this can affect the quality of the monitoring. Parents also requested that the caps be more aesthetic and should come in a range of colours and/or patterns appropriate to newborn care. Parents verbally commented that this was important to them as they would want to be taking photos of, and with, their newborn and the current cap was not appealing.

The feedback from parents confirmed that they found the SurePulse monitoring to be reassuring and that having a wireless system was more convenient and beneficial to them. These positive comments were reflected across parents using the device in the delivery room and within the neonatal unit.

iv. Device Data Analysis

**Figure 4:**
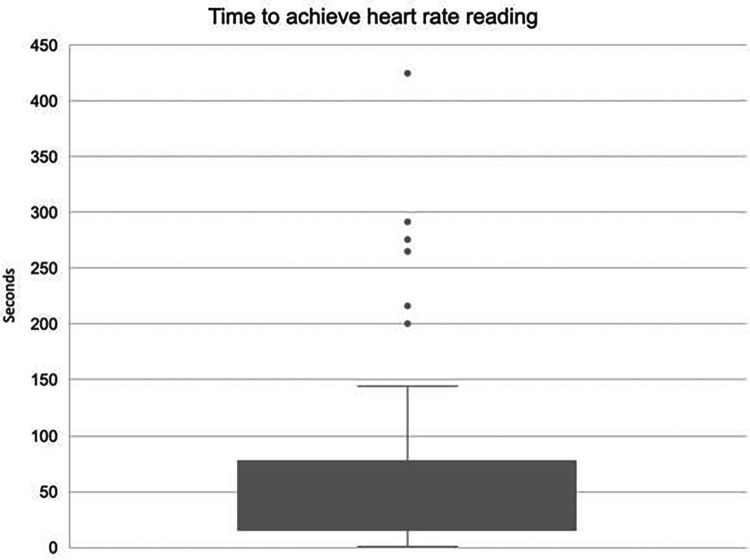
Box and Whisker plot showing visible heart rate acquisition time.

Reliability and consistency of the data from monitoring is paramount to infant safety within neonatal care. Data were downloaded from each deployed study device to evaluate time from application to achieving a numerical heart rate (acquisition time). On average, acquisition of heart rate was a median of 20 s (IQR 15–76 s; Figure 4). There were six infants who were outliers and experienced prolonged acquisition times (>150 s). All these outlier cases occurred in a delivery context with 5 of the 6 cases being term infants (range 34–42 weeks gestation). All outliers were born in good condition. In these outlying cases, users reported issues with module signal and with needing to change cap sizes to better fit the individual infant.

Overall, the heart rate acquisition time was good with 63% of deployments gaining readings in under 60 s and 55% achieving adequate signal within 30 s. This indicates that in the majority of cases the SurePulse device is operating within an acceptable timeframe to be clinically useful.

A range of standard monitoring options were used alongside the study monitor. Selection of standard monitoring varied depending on the clinical situation. For delivery room deployments, standard monitoring included cord palpation, auscultation with a stethoscope and pulse oximetry. For deployments in NICU standard monitoring options were pulse oximetry (PO), electrocardiogram (ECG) or apnoea alarms. There were 33 cases where the method of standard monitoring was not recorded. There were 19 infants who were recorded as receiving ECG alongside SurePulse monitoring. From these 19 cases, HCP's reported that in 58% the study device provided monitoring in a shorter timeframe than ECG monitoring. Pulse oximetry was often applied in conjunction with another form of standard monitoring (ECG or stethoscope auscultation). In these cases of PO monitoring, SurePulse monitoring was perceived to be faster in 40% of infants. Pulse oximetry monitoring is available at each resuscitaire. There was no clarification from the feedback forms as to whether SurePulse or PO monitoring was applied first. This may have affected HCP perception of which monitoring type provided readings more rapidly.

## Discussion

The results of this study must be interpreted in the context that the SurePulse device was applied to well and stable infants in the majority of cases. The device was not utilised in any infants who required significant resuscitation and therefore, we cannot comment on the reliability of the device signal in a compromised infant.

This study shows that the SurePulse device is able to be deployed and provide heart rate monitoring in a clinically acceptable timeframe. The device was rated by both parents and professionals as comfortable for the infant and reliable to use. Wireless monitoring has the potential to allow parents greater autonomy in interacting and handling their baby, improving confidence and facilitating a positive neonatal experience. This type of technological advancement could enhance family integrated care with parents more easily able to provide care to their baby and to position themselves for cuddles in any chair at any angle around the cot-space, rather than, as is the current situation, being limited by monitoring wires. Further evaluation of this monitoring device in neonatal patients with prolonged stays on NICU would be beneficial to ascertain if wireless monitoring would enable more infants to participate in developmentally appropriate activities, such as play and tummy time with parents on neonatal playmats, without compromise of their continuous monitoring.

The current version of the SurePulse device is able to provide reliable heart rate monitoring. This study has shown that healthcare professionals would want additional features in order to elevate this product ahead of current monitoring methods. Desirable features for future development include incorporation of oxygen saturation and temperature monitoring into the device. With these features SurePulse would be an ideal monitoring device for preterm and term infants at delivery and would have significant utility in monitoring neonatal patients in high dependency and special care environments, where wireless monitoring would be particularly advantageous in enabling parental involvement in infant care and addressing a gap for facilitation of family-integrated care.

### Summary

This study indicates that the SurePulse device has potential to be a significant advancement in the way neonatal patients are monitored in a variety of post-delivery circumstances. Staff feedback supports the ease of use and family-friendly care approach that wireless monitoring can provide. The device was originally developed to provide a rapid and accurate method of assessing heart rate at emergency deliveries. As this study has demonstrated this wireless monitoring device offers utility throughout the neonatal journey, not simply in the delivery room.

## Data Availability

The original contributions presented in the study are included in the article/**Supplementary Material**, further inquiries can be directed to the corresponding author.
