# Supplementary material for: A clinical evaluation and acceptability study of the innovative SurePulse VS wireless heart rate monitor across the neonatal journey
Source: Front Pediatr. 2024 May 17;12:1355777. doi: 10.3389/fped.2024.1355777 (PMC11140120; doi:10.3389/fped.2024.1355777)
Supplement: Supplementary file 1 [file Image1.pdf]

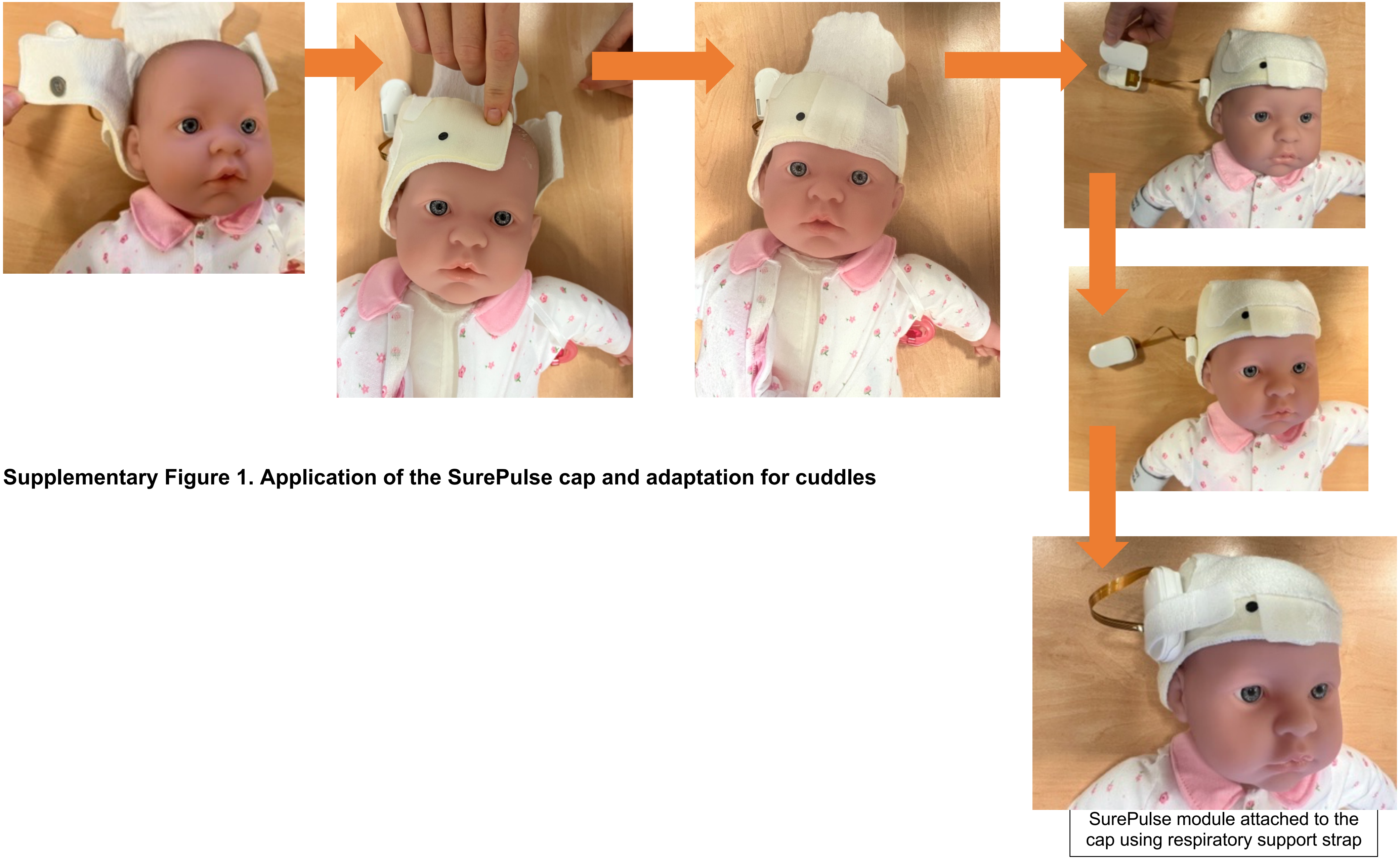


**Supplementary Figure 1. Application of the SurePulse cap and adaptation for cuddles**
